# The Impacts of Intervertebral Disc Degeneration of the Spine, Alcohol Consumption, Smoking Tobacco Products, and Glycemic Disorders on the Expression Profiles of Neurotrophins-3 and -4

**DOI:** 10.3390/biomedicines12020427

**Published:** 2024-02-13

**Authors:** Rafał Staszkiewicz, Dorian Gładysz, Dawid Sobański, Filip Bolechała, Edward Golec, Werner Dammermann, Beniamin Oskar Grabarek

**Affiliations:** 1 Collegium Medicum, WSB University, 41-300 Dabrowa Gornicza, Poland; drdsobanski@gmail.com; 2 Department of Neurosurgery, 5th Military Clinical Hospital, SP ZOZ Polyclinic in Cracow, 30-901 Cracow, Poland; gladyszdorian875@gmail.com; 3 Department of Neurosurgery, Faculty of Medicine in Zabrze, Academy of Silesia, 40-555 Katowice, Poland; 4 Department of Neurosurgery, Szpital sw. Rafala in Cracow, 30-693 Cracow, Poland; 5 Department of Neurosurgery, Faculty of Medicine and Health Sciences, Andrzej Frycz Modrzewski University in Cracow, 30-705 Cracow, Poland; 6 Department of Forensic Medicine, Jagiellonian University Medical College, 31-531 Cracow, Poland; filip.bolechala@uj.edu.pl; 7 Department of Rehabilitation in Orthopaedics, Faculty of Motor Rehabilitation, Bronisław Czech University of Physical Education, 31-571 Cracow, Poland; bgolec@poczta.onet.pl; 8 Center of Internal Medicine II, University Hospital Brandenburg, 03048 Brandenburg, Germany; werner.dammermann@mhb-fontane.de; 9 Faculty of Health Sciences Brandenburg, Brandenburg Medical School Theodor Fontane, 16816 Neuruppin, Germany; 10 Gyncentrum, Laboratory of Molecular Biology and Virology, Department of Molecular Biology, 40-851 Katowice, Poland

**Keywords:** intervertebral disc degeneration, neurotrophins 3 and 4, pain, Pfirrmann scale

## Abstract

In the etiology of discogenic pain, attention is paid to the role of neurotrophic factors, which include classic neurotrophins (NTs). This study aimed to assess changes in the concentrations of NT-3 and NT-4 in the intervertebral discs (IVDs) of the lumbosacral (L/S) spine depending on the advancement of degenerative changes, pain severity, habits, and comorbidities. The study group included 113 patients who underwent microdiscectomy due to degenerative IVD disease of the L/S spine. The severity of degenerative IVD changes was assessed using the five-point Pfirrmann scale, and the pain intensity was assessed according to the visual analog scale (VAS). In turn, the control group included 81 participants from whom IVDs of the L/S section of the spine were collected post-mortem during forensic autopsy or organ donation. At the mRNA level, we noted NT-3 overexpression in the test samples compared with the controls (fold change (FC) = 9.12 ± 0.56; *p* < 0.05), while NT-4 transcriptional activity was decreased in the test samples compared with the controls (FC = 0.33 ± 0.07; *p* < 0.05). However, at the protein level, the concentrations of NT-3 (134 ± 5.78 pg/mL vs. 6.78 ± 1.17 pg/mL; *p* < 0.05) and NT-4 (316.77 ± 8.19 pg/mL vs. 76.92 ± 4.82 pg/mL; *p* < 0.05) were significantly higher in the test samples compared with the control samples. Nevertheless, the concentration of both proteins did not statistically significantly change depending on the advancement of degenerative changes and the pain intensity (*p* > 0.05). In addition, higher levels of NT-3 and NT-4 were noted in IVD samples from patients who consumed alcohol, smoked tobacco, were overweight/obese, or had comorbid diabetes compared with patients without these risk factors (*p* < 0.05). Our analysis confirmed that differences in the degenerative process of IVD, energy metabolism, and lifestyle are related to changes in the concentration profiles of NT-3 and NT-4.

## 1. Introduction

The intervertebral discs (IVDs) are intervertebral cartilage with flat and shallow fibrocartilage located between the facing surfaces of the vertebral bodies [1]. An analysis of the available literature indicates that degenerative IVD disease affects 90% of the general population [2]. Most patients experience this disease asymptomatically [2]. Approximately 85% of cases of IVD degeneration of the L/S spine occur in the L5–S1 space [3]. IVD degeneration can also be associated with age, gender, ethnic origin, the nature of work performed, genetic factors, the type of physical activity, and quality of life [3]. In the etiology of discogenic pain, attention is paid to the role of neurotrophic factors, which include classic neurotrophins (NTs), transforming growth factors, fibroblast growth factors, insulin-like growth factors, platelet-derived growth factors, neuropoietins, and a group of non-neuronal growth factors [4].

NTs are low-molecular-weight growth factors that regulate cellular processes that determine the proper functioning of the central and peripheral nervous system [5,6]. They are characterized by neurotrophic effects, i.e., stimulating and regulating neurogenesis [5,6]. It should be borne in mind that the amount of NTs synthesized and released is only sufficient for a certain number of neurons [5,6].

There are three families of factors with neurotrophic properties, namely, (1) classic NTs, (2) glial-cell-line-derived neurotrophic factor family ligands (GFLs), and (3) neurokines (neuropoietic neurokines). The first identified factor in the neurotrophin family was the nerve growth factor (NGF). This group also includes brain-derived neurotrophic factor (BDNF), NT-3, NT-4, NT-6, and NT-7, the latter two of which have not been identified in mammals [7,8,9].

All NTs are synthesized in the form of biologically inactive precursor proteins. The synthesized NTs contain a signal peptide (pre-protein) and a precursor protein (pro-protein). The formation of the precursor form of neurotrophin (proNT) is conditioned by cutting off the hydrophobic region of the pre-protein molecule at the N-terminus of the amino acid. It is currently believed that NGF and NT-3 arise from the duplication of one common gene, while BDNF and NT-4 come from a different “ancestor gene” [4,6].

The biological effects of NTs result from their interactions with receptors from the tropomyosin-related kinase (Trk) and p75NTR family, which are members of the tumor necrosis factor-alpha (TNF-α) family [10,11,12]. However, all NTs can interact with the p75NTR receptor, which acts as a co-receptor for Trk receptors, or induce other signaling pathways independently of Trk receptors [10,11,12]. The binding of neurotrophin to the Trk receptor, for which it has the greatest affinity, causes the dimerization of the receptor, trans-autophosphorylation, and, consequently, the activation of signaling pathways, including extracellular-signal-regulated kinase (Ras/ERK), phosphatidylinositol 3-kinase (Akt/PI3K), phospholipase C, gamma 1 pathway (PLC-γ1), and mitogen-activated protein kinase (MAPK) [10,11,12]. In turn, the interaction of neurotrophin with the p75NTR receptor results in the activation of nuclear factor kappa B (NFkB) [10,11,12].

This results in a wide spectrum of complex effects of NTs. However, their role and significance in the etiopathogenesis of discogenic pain in the course of degenerative IVD disease is still not sufficiently understood [6].

Moreover, to the best of the authors’ knowledge, no studies have assessed the influences of gender, energy metabolism (i.e., overweight/obesity), lifestyle (i.e., smoking tobacco and alcohol consumption), and glycemic levels on the concentrations of NT-3 and NT-4 in patients with degenerative IVD disease of the L/S spine. Another reason for undertaking research in this area was our previous observations regarding the relationship between the concentrations of brain-derived neurotrophic factor (BDNF), glial-cell-derived neurotrophic factor (GDNF), and growth-associated protein 43 (GAP-43) in IVD samples from patients with degenerative IVD disease compared with control samples [13]. The research conducted so far indicates that smoking tobacco products significantly increases the risk of IVD degeneration [14,15,16,17]. Alcohol consumption has also been shown to adversely affect the development of IVD degeneration [18]. Moreover, alcohol significantly affects the perception of pain [19,20]. Overweight/obesity is another undoubted factor accelerating IVD degeneration [21,22,23,24,25,26,27]. Moreover, overweight/obesity increases the risk of glycemic disorders, including the development of insulin resistance [28,29].

Therefore, this study aimed to assess changes in the concentrations of NT-3 and NT-4 in the IVDs of the L/S section of the spine depending on the advancement of degenerative changes, pain intensity, habits, lifestyle, and comorbidities.

## 2. Materials and Methods

This section builds upon our previous works in [13,30,31].

### 2.1. Ethical Considerations

This research was conducted per the guidelines of the 2013 Declaration of Helsinki on human experimentation. Data confidentiality and patient anonymity were consistently maintained. Patient-identifying information was removed before the database analysis. Informed consent was obtained from all patients. Consent to conduct this study was obtained from the Bioethics Committee operating at the District Medical Chamber in Krakow (no. 162/KBL/OIL/2021).

The collection of postmortem materials for research is regulated by the Act of 1 July 2005, on the collection, storage, and transplantation of cells, tissues, and organs (Journal of Laws of 2020, item 2134) [32].

### 2.2. Study Group

The study group consisted of 113 patients, including 55 women and 58 men, who underwent microdiscectomy due to degenerative IVD disease of the L/S spine. Qualification for the neurosurgical procedure included assessing each patient’s clinical condition, the period of severe IVD pain in the L/S spine (6–12 weeks), assessing the results of magnetic resonance imaging of the L/S spine, and a neurological examination. The degree of advancement of degenerative changes in the km was determined using the 5-point Pfirrmann scale [33]. Each patient was asked to mark the point corresponding to the pain intensity in the L/S spine on the 10-point visual analog scale (VAS). The inclusion and exclusion criteria for the study group are presented in Table 1. They are the same as those shown in the previous works [30,31]. In total, 7 people had a primary education, 59 vocational, 39 secondary, and 8 higher. The details are provided in our previous work [34].

### 2.3. Control Group

The control group included 81 participants, including 53 women and 38 men, from whom IVDs of the L/S section of the spine were collected post-mortem during forensic autopsy or organ donation. The inclusion and exclusion criteria for the study group are presented in Table 2. They are the same as those shown in previous works [30,31].

To verify the absence of degenerative changes in the IVD samples, staining was performed using hematoxylin and eosin (H&E) dyes according to the manufacturer’s recommendations (H&E staining kit; Abcam, Cambridge, MA, USA). Two independent investigators evaluated each slide.

### 2.4. Securing the Collected Material for Molecular Testing

The IVD samples collected from the study and control groups were thoroughly washed to remove blood and then placed in Eppendorf tubes with TRIzol reagent (Invitrogen Life Technologies, Carlsbad, CA, USA) to protect the material for ribonucleic acid extraction and protein isolation. The prepared materials were stored in low-temperature conditions (−80 °C) until the beginning of the molecular analyses.

### 2.5. NT-3 and NT-4 mRNA Expression Profiles in the IVDs of the L/S Spine Obtained from the Study and Control Groups Using the Real-Time Polymerase Chain Reaction Technique Preceded by Reverse Transcription (RT-qPCR)

After qualitative and quantitative analysis of the extracted RNA, RT-qPCR was performed using complementary primer pairs (Genomed, Warsaw, Poland; Table 3). Glyceraldehyde-3-phosphoaldehyde dehydrogenase (GAPDH) was used as an endogenous control for the RT-qPCR reaction. Three technical replicates were performed in 50 µL of the reaction mixture for each biological replicate. The specificity of the RT-qPCR reaction was confirmed by determining the melting temperature (Tm) for each amplimer. Changes in gene expression were normalized relative to mRNA expression (using the 2^−∆∆Ct^ method).

### 2.6. Assessment of the NT-3 and NT-4 Protein Concentration Profiles in the IVDs of the L/S Spine Obtained from the Study and Control Groups

The concentrations of NT-3 and NT-4 in the IVDs of degenerated and healthy patients were determined using an enzyme-linked immunosorbent assay (ELISA) using the anti-NT-3 antibody (Neurotrophin-3 Polyclonal Antibody, BS-0160R, STI, Poznań, Poland) and the anti-NT-4 antibody (Neurotrophin-4 Polyclonal Antibody, BS-0158R, STI, Poznań, Poland), according to the manufacturer’s protocol. GAPDH was used as the endogenous control in both assays (Santa Cruz Biotech, Dallas, TX, USA).

A detailed protocol for performing ELISA and Western blot tests has been provided in previous works [30,31].

### 2.7. Statistical Analysis

The statistical analysis of the results was performed assuming a threshold of statistical significance of *p* < 0.05 in the Statistica 13 PL program (Statsoft, Kraków, Poland). 

The Shapiro–Wilk test was used to assess the compliance of the data distribution with the normal distribution, which was confirmed. Therefore, parametric methods, i.e., Student’s *t*-test for independent groups or one-way ANOVA and Tukey’s post hoc test, were used in the next stage of the statistical analysis. The homogeneity of variances was checked using Levene’s test.

## 3. Results

### 3.1. Expression Changes in the mRNA and Protein Levels of NT-3 and NT-4 in Control and Test Samples

**Figure 1:**
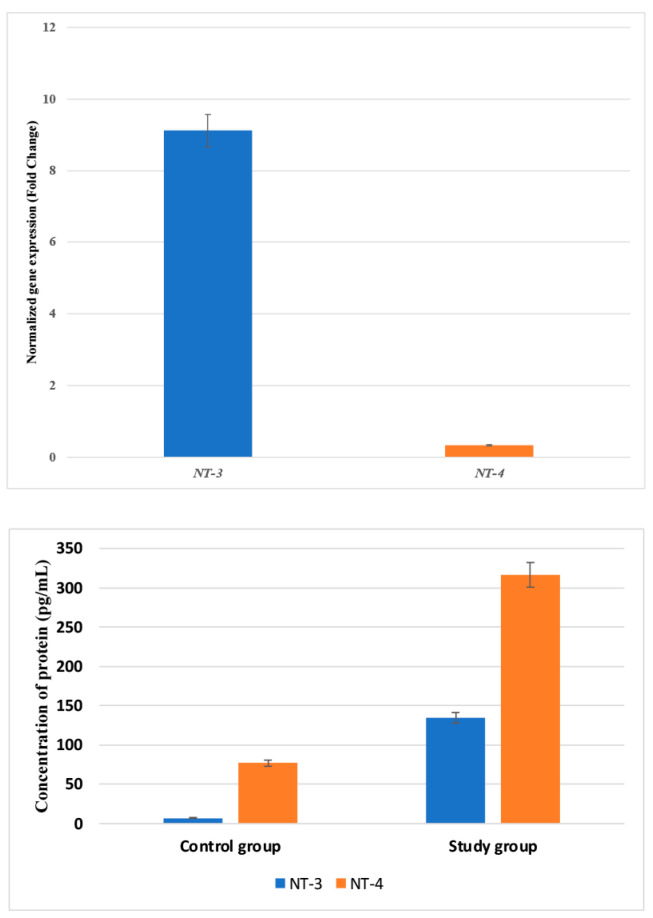
Variances in the expression patterns of *NT-3* and *NT-4* at the mRNA and protein levels in the study and control groups. NT-3, neurotrophin-3; NT-4, neurotrophin-4. Data are presented as means with standard deviation.

First, the expression patterns of the NT-3 and NT-4 mRNA and the proteins they encode were compared between the examined and control samples. The overexpression of *NT-3* was noted in the tested samples compared with the control samples, both at the mRNA and protein levels (Figure 1; *p* < 0.05). In turn, a decrease in *NT-4* expression at the mRNA level was demonstrated in the tested samples, along with a significantly higher concentration of NT-4 protein, compared with the control samples (Figure 1; *p* < 0.05).

### 3.2. Concentrations of mRNA and Protein of NT-3 and NT-4 in Control and Examined Samples, Considering the Stage of Radiological Degenerative Changes in IVDs according to the Pfirrmann Scale

The IVD samples were assessed depending on the radiological advancement of the degenerative changes (Table 3). Based on the statistical analysis, statistically significant differences were noted in the expressions of *NT-3* and *NT-4* mRNA and proteins depending on the stage of degenerative changes (Table 4; *p* < 0.05), with the highest NT-3 expression observed in IVD samples representing stage 3 lesions according to the Pfirrmann scale (Table 4; *p* < 0.05).

The silencing of the transcriptional activity of *NT-4* was demonstrated at the mRNA level, with the lowest expression for this transcript recorded in samples representing grades 2 and 4 of radiological advancement of lesions according to the Pfirrmann scale (Table 4). At the protein level, the highest concentration of NT-4 was found in IVD samples representing stage 4 degenerative changes according to the Pfirrmann scale (Table 4; *p* < 0.05).

The expressions of NT-3 and NT-4 at both the mRNA and protein levels did not differ significantly between IVD samples with degeneration grades of 3 and 4 according to the Pfirrmann scale (Table 4; *p* > 0.05).

### 3.3. The Concentrations of mRNA and Protein of NT-3 and NT-4 in the Tested Samples Depending on the Pain Degree Measured with the VAS

Then, we assessed whether the expressions of NT-3 and NT-4 at the mRNA and protein levels significantly changed depending on the pain severity (Table 5). The analysis of variance (ANOVA) did not show significant changes in the *NT-3* and *NT-4* mRNA expression profiles depending on the pain intensity measured with the VAS (Table 5; *p* > 0.05). Overexpression of *NT-3* mRNA was noted, regardless of the pain intensity, while expression silencing was found for *NT-4 mRNA*.

The ANOVA did not show significant changes in the concentration of both proteins depending on the degree of pain experienced by the patient (Table 3; *p* > 0.05). However, the highest concentration of NT-3 was recorded in the group of patients declaring a pain intensity at level 5, and in the case of NT-4, at levels 5 and 6 (Table 3). Moreover, the concentration of NT-3 could not be determined for pain intensities of grades 2 and 3, and the concentration of NT-4 was below the detection threshold for grades 2–4 according to the VAS (Table 5; *p* > 0.05).

### 3.4. Variances in the Expression Profiles of NT-3 and NT-4 at the mRNA and Protein Levels in IVD Samples Obtained from the Study and Control Groups

In the next step, we analyzed whether the expression profiles of NT-3 and NT-4 at the mRNA and protein levels were dependent on gender, BMI, co-occurrence of diabetes, alcohol consumption, and smoking (Table 6; *p* < 0.05). We noted significantly higher expressions of both analyzed NTs in obese compared with overweight (Table 6; *p* < 0.05) and normal-body-weight (Table 6; *p* < 0.05) patients; patients with concomitant diabetes (Table 6; *p* < 0.05); patients consuming alcohol compared with those who did not consume it (Table 6; *p* < 0.05); and patients smoking tobacco products compared with those who did not declare smoking tobacco (Table 6; *p* < 0.05). We did not confirm whether the expressions of NT-3 and NT-4 in patients with IVD degeneration were gender-dependent (Table 6; *p* > 0.05).

### 3.5. Regression Analysis of Variables Potentially Associated with BDNF, GDNF, and GAP-43 Levels in IVDs and Serum Samples from the Study Groups

In the final step, linear and multiple regression analyses of the clinical variables were performed to determine the associations with NT-3 and NT-4 expression, as shown in Table 7. A linear regression analysis considering one factor (predictor—age) showed no effect on the expressions of the NT-3 and NT-4 studied. In contrast, both regression analyses considering one or several predictors found the concentrations of NT-3 and NT-4 to be correlated with BMI, smoking, and alcohol consumption (Table 7).

## 4. Discussion

Increased NT expression in severely degenerated IVDs has been associated with chronic low back pain associated with the progression of disc degeneration [35].

It has been suggested that the involvement of NT-3 and NT-4 in the etiopathogenesis of degenerative IVD disease and the development and progression of the accompanying pain [36] is related to the signal transduction initiated by the interaction of NT-3 or NT-4 with TrkA, the TrkB receptor, TrkC, or p75NTR [37]. Given these observations, it can be assumed that NT-3 and NT-4 do not directly affect the occurrence of discogenic pain and the development of IVD degenerative disease of the L/S spine; rather, they exert an indirect effect, acting as activators of several signaling pathways and thereby changing the expression profiles of other neurotrophic factors that are more important in the neo-inversion process.

So far, research focusing on NT-3 and NT-4 has focused on improving the understanding of these NTs and the possibility of their therapeutic use in demyelinating and neurodegenerative diseases, depression, and mood disorders [38]. This observation is important in the context of degenerative IVD because patients with depression and anxiety disorders tend to somatize their symptoms, feeling them more than their clinical condition would indicate. 

Therefore, depression and anxiety are associated with an increased perception of pain intensity, while a prolonged duration of acute pain leads to worsened mood [39,40]. This may be a reason for the lack of statistically significant changes in the NT-3 concentration depending on the degree of pain.

Nevertheless, the involvement of NT-3 in the etiology of discogenic pain is probable considering the high expression of the TrkC receptor demonstrated in neurons of the corticospinal tract, to which only NT-3 has affinity among NTs. However, this requires additional research [41,42]. 

Sahenk et al. [43] assessed the possibility of using NT-3 applied to muscle cells using an adenovirus vector in the treatment of Charcot–Marie–Tooth neuropathy [43]. The researchers obtained promising results in the field of peripheral nerve regeneration, although further analyses and research are necessary [43].

Research conducted by Omura et al. [44] indicates that the expression levels of BDNF, NT-3, and NT-4 vary depending on the type of damage that led to nerve damage [44].

NT-3 expression did not undergo any statistically significant changes, regardless of the cause of sciatic nerve damage, while the NT-4 level decreased after sciatic nerve transection [44]. Wang et al. [45] indicated that NT-4 may be a new effective drug with anti-inflammatory effects in the central nervous system by modulating the TrkB/PI3K/Akt/forkhead box protein O1 (FoxO1) pathway [45]. In addition, Omar et al. showed that they are crucial factors in the pathogenesis of neurological diseases and can also constitute new therapeutic targets in diseases of the central nervous system resulting from trauma, nerve degeneration, and the disruption of blood flow through blood vessels [46].

Moreover, we observed silencing of NT-4 expression at the mRNA level with simultaneous overexpression in IVDs obtained from patients in the study group compared with control samples. This may be due to several reasons, including the simultaneous influences of various factors on the transcript as well as their synergistic effects. Transcription and translation processes take place in different cell compartments, and both processes are not strictly synchronized [47,48]. Moreover, these processes are interconnected because the resulting mRNA molecule is a template for protein synthesis [49,50,51]. After transcription, mRNA can be translated many times [52,53], which may partially explain our results. It is also possible that the resulting *NT-3* and *NT-4* transcripts are degraded by, for example, microRNA (miRNA) molecules showing incomplete complementarity to the target mRNA, which reduces the mRNA pool constituting the template for protein synthesis [54,55]. Another potential reason for the observed changes may be the potentially extended half-lives of NT-3 and NT-4 proteins, which allow them to accumulate in the cells even when expression at the mRNA level is silenced, although this has not yet been assessed.

A novel aspect of our research Is the assessment of the expression profiles of NT-3 and NT-4 in patients with IVD degeneration, including metabolic disorders and habits. Some observations indicate that glycemic disorders and alcohol consumption influence the expression of these NTs. Ihara et al. reported a decrease in NT-3 mRNA expression in the muscle tissue of rats with pharmacologically induced diabetes compared with rats without induced diabetes. The researchers suggested that the reduction in NT-3’s transcriptional activity may be a marker of early damage to sensory neurons [56]. The research conducted by Li et al. produced compelling results regarding the influences of alcohol and tobacco consumption on the expressions of NT-3 and NT-4 [57]. Li et al. showed an increase in the expressions of NT-4 and BDNF in a group of rats treated with naloxone, an opioid receptor antagonist that reverses the action of opioids and abolishes the reactions caused by them, with a simultaneous decrease in the expressions of NT-4 and BDNF in rats administered heroin compared with the control. The observed changes in the expression profiles of BDNF and NT-4 in this group of rats indicate that the mentioned neurotrophic factors have a neuroprotective effect on the nucleus accumbens (nAc) in the event of sudden cessation of chronic heroin use. Furthermore, an increase in the expression of NT-3 was noted in the group of rats addicted to heroin, alongside a decrease in expression in rats administered naloxone, which indicates the activation of adaptive mechanisms and the nAc striving for homeostasis [58]. Moreover, a significant increase in NT-3 expression was found in the hippocampus after chronic exposure to ethanol [59], which is consistent with our observations and may suggest that patients declaring alcohol consumption who have been drinking for a long time may have developed an addiction syndrome [59]. This hypothesis is confirmed by the observations of Requena-Ocaña et al., who found that BDNF and NT-3 act as factors compensating for cognitive impairment in the early stages of alcohol addiction, although these properties are lost at later stages. The NT-3 expression profile also depended on education in alcohol-dependent patients, i.e., NT-3 expression was decreased in patients with higher education, while NT-3 was overexpressed in people without higher education. This is likely due to impaired neurogenesis [60] and is consistent with our observations, where the majority of patients did not have higher education [34]. This is also confirmed by the research of Silva-Peña et al., who linked low education and alcohol abuse with reduced NT-3 and BDNF concentrations [61]. In addition, the research by Yang et al. indicates that smoking tobacco products is associated with reduced NT-3 expression in the rat hippocampus [62]. In contrast with the pattern reported by Yang et al. [62], we noted an overexpressed NT-3 expression profile in degenerated IVD samples obtained from smokers in our study. Kimata et al. noted an increase in the expressions of NT-3 and NT-4 in the tears of atopic keratoconjunctivitis (AKC) patients who were passive smokers compared with a group of passive smokers without AKC and healthy volunteers, suggesting that passive smoking increases the expressions of NT-3 and NT-4 in tears, which, in turn, may exacerbate AKC. Moreover, significant changes in the expressions of these NTs occurred only in AKC patients who were passive smokers, indicating that AKC was the factor determining the changes in the expression patterns of NT-3 and NT-4 and the associated inflammation [63]. This suggests that although we found overexpression of NT-3 and NT-4 among patients with degenerative IVD disease who used tobacco products in our study, the main factor causing the changes in NT-3 and NT-4 expression is the degenerative IVD process, not just smoking.

Based on the study of the NT-3 and NT-4 concentration profiles, their involvement in the etiology of degenerative IVD disease of the L/S spine section seems probable, although further analyses are necessary. In addition, after a comparative analysis between our results and studies by other authors, it can be concluded that habits and lifestyles are associated with degenerative changes in IVDs. Our multiple regression analysis confirmed this.

In light of our thorough analysis of the existing literature, it becomes evident that our investigation into the expression profile of NT-3 and NT-4 within the degenerative disease IVD of the L/S spine marks a pioneering endeavor in this field. This study stands out for its innovative approach and significant contribution to understanding the molecular mechanisms underlying such conditions. Our findings not only add valuable insights but also set a new standard for future research in this area.

Our research has limitations. Firstly, comparing the obtained results with the observations of other researchers, particularly regarding the influences of lifestyle and habits on the expression profiles of NT-3 and NT-4, it is necessary to consider performing brain imaging and combining the obtained expression results with sociodemographic data. Moreover, the groups compared should be equal in number, especially concerning habits. Thirdly, other methods could be used to assess the expressions of NT-3 and NT-4, as well as to investigate and determine the mechanisms that modulate NT-3 and NT-4 expression.

## 5. Conclusions

Given the results of our research and the observations of other researchers, it seems that NT-3 and NT-4 do not directly affect the occurrence of discogenic pain and the development of degenerative IVD disease of the L/S spine; rather, they have an indirect effect, functioning as activators of several signaling pathways, which results in changes in the expression profiles of other more important neurotrophic factors in the neo-inversion process. Moreover, the results obtained in our study indicate that the degenerative process within the IVDs is the main factor determining the changes in NT-3 and NT-4 expression, not the use of tobacco products or alcohol consumption, although these factors accelerate the development of degenerative spine disease. However, further research is necessary.

## Figures and Tables

**Table 1:** Inclusion and exclusion criteria for the study group.

Inclusion Criteria	Exclusion Criteria
Aged > 18 years old	Aged < 18 years old
Lumbosacral spine-isolated IVD degeneration of a prolapse/extrusion character determined using magnetic resonance imaging	IVD degeneration in the lumbosacral spine of a protrusion or sequestration character determined using magnetic resonance imaging
Discogenic pain and/or symptomatic sciatica without improvement after non-surgical treatment for at least 6 weeks	Previous surgical procedures due to IVD degeneration in the lumbosacral spine
No other coexisting pathologies of the spine	Inflammatory and autoimmune diseases
	Condition after spine injury
	Dementia/mental disorders
	Polyneuropathy
	Pregnancy
	Coexisting diseases, including metabolic diseases
	Neoplasms: metastatic tumors in the spine; lymphoma; leukemia; spinal cord tumors; retroperitoneal tumors; and primary shaft tumors
	Inflammatory diseases: inflammation of the bone elements of the spine
	Osteoporosis
Disease duration of no longer than 12 weeks	Disease duration exceeding 12 weeks or of less than 6 weeks
	Inflammatory disease history

**Table 2:** Inclusion and exclusion criteria for the control group.

Inclusion Criteria	Exclusion Criteria
Up to 45 years of age	Over 45 years of age
No signs of degeneration in the collected material during microscopic examination	Features of degeneration in the collected material during microscopic examination (hematoxylin and eosin staining)
No neoplastic disease history	Neoplastic disease history
No inflammatory diseases: inflammation of the bone elements of the spine (osteomyelitis); IVD inflammation; epidural empyema; shingles; arthritis; inflammatory infiltrates of the rectum; Scheuermann’s disease; and Paget’s disease	Spinal disease history

**Table 3:** Nucleotide sequences of primers used in the RT-qPCR reaction for NT-3, NT-4, and GAPDH mRNA.

mRNA	Oligonucleotide Sequence
*NT-3*	Forward: 5′-CGTGGTGGCGAACAGAACAT-3′ Reverse 5′-GGCCGATGACTTGTCGGTC-3′
*NT-4*	Forward: 5′-CTGTGTGCGATGCAGTCAGT-3′ Reverse 5′-GCAGCGGGTTTCAAAGAAGT-3′
*GAPDH*	Forward: 5′-GGTGAAGGTCGGAGTCAACGGA-3′ Reverse 5′-GAGGGATCTCGCTCCTGGAAGA-3′

Forward, sensible starter; reverse, antisense primer; GAPDH, dehydrogenase 3-phosphoglyceraldehyde; NT-3, neurotrophin-3; and NT-4, neurotrophin-4.

**Table 4:** Concentrations of NT-3 and NT-4 in intervertebral discs of the L/S section taken from the study and control groups determined using an ELISA test.

Neurotrophin	The Advancement of Changes	Fold Change (mRNA)	Protein Concentration (pg/mL)
NT-3	Pfirrmann 2	7.21 ± 1.98 ^a^	45.98 ± 7.18 ^a^
	Pfirrmann 3	11/12 ± 1/17	245.98 ± 9.87
	Pfirrmann 4	10.9 ± 1.76 ^b,c,d,e^	202.98 ± 13.12 ^b,c,d,e^
	Pfirrmann 5	7.26 ± 2.87	43.91 ± 4.87
NT-4	Pfirrmann 2	0.26 ± 0.03	187.98 ± 13.23
	Pfirrmann 3	0.43 ± 0.08	456.99 ± 13.41
	Pfirrmann 4	0.42 ± 0.11	498.18 ± 10.87
	Pfirrmann 5	0.22 ± 0.04	123.91 ± 8.76

NT-3, neurotrophin-3; NT-4, neurotrophin-4; ^a^, statistically significant difference in mRNA and protein expression between the Pfirrmann 2 and Pfirrmann 3 groups (*p* < 0.05); ^b^, statistically significant difference in protein concentration between the Pfirrmann 4 and Pfirrmann 5 groups (*p* < 0.05); ^c^, statistically significant difference in protein concentration between the Pfirrmann 2 and Pfirrmann 4 groups (*p* < 0.05); ^d^, statistically significant difference in protein concentration between the Pfirrmann 2 and Pfirrmann 5 groups (*p* < 0.05); ^e^, statistically significant difference in protein concentration between the Pfirrmann 3 and Pfirrmann 5 groups (*p* < 0.05). Results are presented as means ± standard deviation.

**Table 5:** Concentrations of NT-3 and NT-4 in the intervertebral discs of the L/S section affected by the degenerative process depending on the degree of pain felt.

Neurotrophin	Pain Intensity on the VAS	Fold Change (mRNA)	Protein Concentration (pg/mL)	ANOVA (*p*) ^b^
NT-3	2	3/11	<corner detection	0.114 ^a^ 0.654 ^b^
	3	2.98	<corner detection	
	4	14.76	134.98 ± 4.65	
	5	16.78	201.12 ± 5.67	
	6	7/98	31.87 ± 5.67	
	7	8.76	8.98 ± 2.34	
	8	8/98	8.91 ± 2.91	
	9	9.65	14.98 ± 3.45	
	10	9/12	23.19 ± 6.78	
NT-4	2	0.41	<corner detection	0.118 ^a^ 0.787 ^b^
	3	0.23	<corner detection	
	4	0.21	<corner detection	
	5	0.62	419.98 ± 21.54	
	6	0.61	408.17 ± 18.17	
	7	0.25	210.87 ± 6.76	
	8	0.27	187.98 ± 12.87	
	9	0.26	203.13 ± 10.98	
	10	0.15	89.09 ± 13.87	

NT-3, neurotrophin-3; NT-4, neurotrophin-4; ^a^, *p*-value for ANOVA analysis at mRNA level; ^b^, *p*-value for ANOVA analysis at the protein level.

**Table 6:** The expressions of NT-3 and NT-4 at the mRNA and protein levels in IVD samples obtained from the study group.

Protein	Comparison		mRNA	Student’s t-Test ^1^ or ANOVA^2^ (Study Group)	Protein	Student’s t-Test ^1^ or ANOVA^2^ (Control Group)
NT-3	Gender	Female (n = 43)	9/19 ± 1/12	0.761 ^1^	0.36 ± 0.04	0.981 ^1^
		Male (n = 38)	9/04 ± 0.97		0.39 ± 0.05	
	BMI (kg/m^2^)	Normal (n = 54)	7.29 ±1.54	0.009 ^2^	0.12 ± 0.05	<0.0001 ^2^
		Overweight (n = 42)	8/08 ±1/23		0.40 ± 0.09	
		Obesity (n = 17)	12.00 ± 1.54		0.47 ± 0.09	
	Diabetes	No (n = 91)	5.78 ± 1.01	<0.0001 ^1^	0.23 ± 0.10	0.023 ^1^
		Yes (n = 22)	12.45 ± 2.02		0.43 ± 0.08	
	Smoking	No (n = 77)	8/11 ± 2/12	<0.008 ^1^	0.42 ± 0.11	0.004 ^1^
		Yes (n =36)	10.12 ± 2.34		0.23 ± 0.07	
	Drinking alcohol	No (n = 8)	11.77 ± 1.77	0.876 ^1^	0.52 ± 0.12	<0.0001 ^1^
		Yes (n = 105)	6.89 ± 1.21		0.13 ± 0.06	
NT-4	Gender	Female (n = 43)	135.65 ± 5.17	0.765 ^1^	320.87 ± 11.09	0.376 ^1^
		Male (n = 38)	133.76 ± 5.34		312.67 ± 8.12	
	BMI (kg/m^2^)	Normal (n = 54)	116.06 ± 5.08	<0.0001 ^2^	288.22 ± 9.31	0.009 ^2^
		Overweight (n = 42)	139.97 ± 7.12		319.17 ± 10.19	
		Obesity (n = 17)	148.11 ± 3.19		342.91 ± 6.87	
	Diabetes	No (n = 91)	118.60 ± 2.01	<0.0001 ^1^	288.01 ± 4.13	<0.0001 ^1^
		Yes (n = 22)	150.84 ± 1.76		345.53 ± 5.28	
	Smoking	No (n = 77)	120.19 ± 2.76	<0.0001 ^1^	281.75 ± 0.43	<0.0001 ^1^
		Yes (n =36)	149.22 ± 4.54		351.79 ± 0.65	
	Drinking alcohol	No (n = 8)	135.32 ± 3.87	0.049 ^1^	281.16 ± 8.41	<0.0001 ^1^
		Yes (n = 105)	134.11 ± 2.19		352.37 ± 9.18	

Data are presented as means and standard deviations. BMI, body mass index; NT-3, neurotrophin-3; NT-4, neurotrophin-4. ^1^, *p*-value from the Student’s t-Test; ^2^, *p*-value from the one-way ANOVA analysis.

**Table 7:** Univariate and multivariate regression analyses of the variables that may be associated with NT-3 and NT-4 levels determined in the degenerative IVDs.

Neurotrophin	Characteristic	Expression Level	Linear Regression			Multiple Regression	
			r	R^2^	*p*-Value	Coefficient	*p*-Value
NT-3	Sex	mRNA	0.31	0.07	0.231		
		Protein	0.29	0.09	0.276		
	BMI (kg/m^2^)	mRNA	0.79	0.61	<0.0001	0.3411	<0.0001
		Protein	0.80	0.65	<0.0001	0.408	<0.0001
	Diabetes	mRNA	0.71	0.76	<0.0001	0.321	0.015
		Protein	0.76	0.74	<0.0001	0.308	0.021
	Smoking	mRNA	0.87	0.45	0.001	0.476	0.024
		Protein	0.89	0.41	0.003	0.487	0.021
	Drinking alcohol	mRNA	0.56	0.39	0.024	0.234	0.028
		Protein	0.54	0.42	0.023	0.265	0.029
NT-4	Sex	mRNA	0.21	0.02	0.421		
		Protein	0.18	0.02	0.476		
	BMI (kg/m^2^)	mRNA	0.81	0.87	<0.0001	0.410	0.018
		Protein	0.78	0.81	<0.0001	0.421	0.021
	Diabetes	mRNA	0.56	0.39	<0.0001	0.318	0.017
		Protein	0.56	0.38	<0.0001	0.321	0.021
	Smoking	mRNA	0.80	0.45	0.009	0.309	0.024
		Protein	0.81	0.54	0.003	0.319	0.029
	Drinking alcohol	mRNA	0.54	0.21	0.031	0.151	0.017
		Protein	0.41	0.23	0.029	0.168	0.020

NT-3, neurotrophin-3; NT-4, neurotrophin-4; BMI, body mass index; r, correlation coefficient; IVD, intervertebral disc degeneration. Variables found to be insignificant using linear regression were not included in the multiple regression model.

## Data Availability

The data used to support this study’s findings are included in this article. The data will not be shared due to third-party rights and commercial confidentiality.
